# The Role of Perceived Inter-Ethnic Classroom Climate in Adolescents’ Engagement in Ethnic Victimization: For Whom Does it Work?

**DOI:** 10.1007/s10964-020-01228-8

**Published:** 2020-03-31

**Authors:** Sevgi Bayram Özdemir, Metin Özdemir

**Affiliations:** Center for Lifespan Developmental Research, School of Law, Psychology and Social Work, Örebro University, 701 82 Örebro, Sweden

## Abstract

Immigrant and minority youth are at risk of experiencing victimization due to their ethnic, cultural, or religious background. Despite an increasing number of studies that aims at understanding the consequences of being the target of such negative experiences, little attention has been paid to the factors that might counteract the occurrence of ethnic victimization. The present study aimed to address this gap in knowledge by investigating the possible role of school context. Specifically, the present study examined the extent to which perceived positive contact norms in class and teachers’ reactions to ethnic victimization are linked to engagement in ethnic victimization. It also examined whether such links differ across adolescents with different levels of tolerance toward immigrants. The sample included 963 adolescents residing in Sweden (*M*_*age*_ = 13.11, SD = 0.41; 46% girls). The results showed that perceived positive contact norms in class were associated with a lower likelihood of engagement in ethnic victimization across youth with different levels of tolerance toward immigrants. When adolescents perceived their teachers as not tolerating ethnic victimization, those with high levels of tolerance were less likely to engage in it. However, teacher reactions did not affect the behaviors of adolescents with low and moderate levels of tolerance toward immigrants. The findings indicate the importance of classroom context and teachers in counteracting negative interactions among students of diverse backgrounds.

## Introduction

Immigrant and minority youth are at risk of experiencing unfair treatment and victimization due to their ethnic or cultural background, their religion, or the language they speak. For instance, a recent large-scale study (*N* = 3305) in the U.S. found that 12% of students (from grades 5 through 12) reported being repeatedly targeted by bullies due to their ethnic background (Mendez et al. 2016). Similarly, a study in the Netherlands found that 33 to 42% of ethnic-minority children mentioned being victims of racist name-calling, and 26 to 30% reported experience of ethnic exclusion in school (Verkuyten and Thijs 2002). These negative experiences have detrimental consequences for immigrant and minority youth’s psychosocial (Priest et al. 2013), behavioral (Bayram Özdemir et al. 2019), and school adjustment (Bayram Özdemir and Stattin 2014), and may jeopardize their integration into the host society (Marks et al. 2015). Despite the existence of substantial evidence showing the harmful consequences of ethnicity-based negative treatments, very limited knowledge is available with regard to the factors that might restrain adolescents from victimizing (or provoke them into victimizing) their peers due to their ethnic or cultural background (Bayram Özdemir et al. 2018; Caravita et al. 2019). There is a need to identify the factors that might counteract the occurrence of ethnic victimization (which is defined as the use of derogatory comments or engagement in exclusionary behaviors based on ethnic background). To address this gap in knowledge, the present study examined the extent to which perceived positive contact norms in class and teachers’ reactions to ethnic victimization are linked to engagement in ethnicity-based negative treatments of peers. It also examined whether such links differ across adolescents with different levels of tolerance toward immigrants. Based on previous literature (Cote and Erickson 2009), tolerance was operationalized as comprised of both positive attitudes (i.e., an understanding and endorsement of equality between immigrants and non-immigrants) and positive feelings toward immigrants (i.e., affective evaluations of immigrants) in the present study.

### Positive Inter-Ethnic Contact Norms in Class

Promoting social contact and cooperation between people of different backgrounds is regarded as the base for the development of positive intergroup attitudes and relationships (Allport 1954). Supporting this conceptual argument, a large body of empirical research has shown that the more cross-ethnic friendships adolescents form, the more positive intergroup attitudes they will hold (e.g., Davies et al. 2011; Pettigrew and Tropp 2006). In addition to direct personal contact, such as cross-ethnic friendship, social norms that support positive contact and cooperation between students of diverse cultural affiliations in classroom or school settings may determine young people’s inter-ethnic attitudes and behaviors (Christ et al. 2014). Specifically, as highlighted by social norms theory (Perkins and Berkowitz 1986), young people may display behaviors that are in line with the social norms of the context, and try to avoid contradicting group norms and any consequent social sanctions. Norms supporting contact and cooperation might motivate youth to develop positive views on others who are different than themselves and to engage in contact with outgroup members. In line with these conceptual arguments, a growing body of research has shown that perceived positive contact norms in class (e.g., being inclusive, respecting each other, cooperating in class activities) are associated with lower prejudiced beliefs (Molina and Wittig 2006), more openness to diversity (Schachner et al. in press), greater intercultural competence (Schwarzenthal et al. 2019), and a higher likelihood of forming interethnic friendships (Schachner et al. 2015). Importantly, perceived positive contact norms in class have also been found to be associated with lower perceived discrimination (Schachner et al. in press) and a greater out-group orientation (Schwarzenthal et al. 2018) among immigrant students. Together, these findings suggest that adolescents are influenced by the norms or behaviors of others in their social context, entailing that prejudicial beliefs or inclusive attitudes may also be a function of where youth are situated contextually.

Despite an increasing number of studies examining the potential role of perceived positive contact norms in intergroup relationships, a majority of these studies (with some exceptions; e.g., Schachner et al. 2015) have focused on whether these norms are linked to intergroup attitudes (e.g., prejudiced beliefs or cultural competence). Limited knowledge is available regarding whether and how these norms are associated with adolescents’ actual behaviors, including engagement in ethnic victimization. More importantly, heterogeneity among adolescents has not been thoroughly considered. That is, it is unknown for whom positive contact norms are most influential. In his interactional model of human development, Magnusson (1990) stressed that individual functioning and development cannot be fully understood by adopting either a context-free individual-focused or a purely context-guided approach. Rather, a holistic approach, emphasizing the effects of multiple factors on human behavior and development, is needed to give more complete insight into the complexity of the “whole person.” In line with this argument, the present study examined whether the effect of positive inter-ethnic contact norms in class varies across youth with different attitudes and feelings toward immigrants.

Two competing theoretical perspectives can be adopted to explain how the effect of positive inter-ethnic contact norms in class on engagement in ethnic victimization might vary according to youth’s attitudes and feelings toward immigrants. The first theoretical perspective relies on the *rich-get-richer* hypothesis, which suggests that an optimal social context is more beneficial for individuals with high social capital (Merton 1968). Across several studies, it has been shown that young people with positive attitudes or feelings toward immigrants are more open to diversity and form cross-ethnic friendships (e.g., Özdemir and Bayram Özdemir 2017). When these adolescents are in a class where students respect each other and cooperate in class activities regardless of their ethnic background, they might be more receptive to social norms given that these norms are very much in line with their personal attitudes and feelings about diversity. Relatedly, these adolescents’ likelihood of engagement in ethnic victimization might become even lower.

The second theoretical perspective relies on the *buffering effect* hypothesis, which suggests that social resources may have more of a beneficial effect on children and youth who are more at risk (Cohens et al. 1985). Youth with low tolerance or high prejudiced beliefs often negatively stereotype differences between their own in-group and out-groups. These youth are also at risk of engagement in ethnic victimization (e.g., Bayram Özdemir et al. 2016; Caravita et al. 2019), especially if they are situated in a situatable social context (Bayram Özdemir et al. 2018). When the norms in a social context are not in line with these youth’s attitudes and feelings, they may feel hesitant to act out negatively toward their immigrant peers, so as to avoid social sanctions. That is, positive inter-ethnic contact norms in a class might be particularly effective in suppressing the occurrence of ethnic victimization among youth who are at risk, i.e., those who are low in positive attitudes and feelings toward immigrants.

### Teachers’ Responses to Ethnic Victimization

Teachers are in contact with students of diverse backgrounds on a day-to-day basis. They have the opportunity to oversee interactions among children and youth, and to influence students’ understanding of and approach to others of diverse backgrounds. They can accomplish this in many different ways: being a good role model by valuing diversity and treating students fairly, communicating expected behavioral and social behaviors to students, and generating opportunities for children to cooperate with and learn from each other. In line with these arguments, in a recent study focusing on primary school children in the Netherlands, Geerlings et al. (2017) showed that students who perceived their teacher as displaying more positive norms about multiculturalism (e.g., emphasizing equality, and respecting different cultural views and perspectives) demonstrated more positive outgroup attitudes.

Teachers may not only promote positive interactions among students but also have the opportunity to reduce the occurrence of negative interactions among youth of diverse backgrounds. A limited but growing body of research has provided evidence to support this idea. For instance, in their study of secondary-school students (aged 12–17) in Canada, Closson et al. 2014 found that students report low levels of ethnic discrimination in schools where teachers value and support diversity (Closson et al. 2014). Similarly, Verkuyten and Thijs (2002) reported a negative association between experience of racist victimization and perceived teachers’ reaction to ethnic victimization among primary school students in the Netherlands. Specifically, fewer children reported the experience of racist bullying when they believed that they could tell their teacher about it and that the teacher would react. Together, these studies suggest that teachers’ diversity beliefs and their reactions to ethnic victimization may reduce the occurrence of ethnic-victimization experiences.

One of the main limitations of the existing literature on teachers’ beliefs and behaviors is that ethnic victimization has generally been examined from the perspective of victims (e.g., How often have you had experience of someone treating your racial or ethnic group as inferior?). Thus, there is only limited knowledge on whether teachers’ reactions to ethnic victimization also effectively eliminate the behaviors of perpetrators. Additionally, it is unknown whether teachers’ reactions to ethnic victimization work equally well for all adolescents. Two competing theoretical perspectives that were described previously (as the *rich-get-richer* hypothesis and the *buffering effect* hypothesis) can also be used here to explain how the effect of teachers’ reactions to ethnic victimization might vary among adolescents with different attitudes and feelings toward immigrants. On the one hand, based on the premises of the *rich-get-richer* hypothesis (Merton 1968), it can be argued that tolerant youth have high social capital to start with. When teachers expound the view that no-one can make negative comments about others because of their background, these young people might be more receptive to this message given that it is in line with their own personal views and feelings about immigrants. On the other hand, on the basis of the *buffering effect* hypothesis (Cohens et al. 1985), optimal social context (i.e., no-tolerance to ethnic victimization in class) might be expected to prevent adolescents at risk (i.e., those who are in low in positive attitudes and feelings toward immigrants) acting out in line with their views and feelings.

## The Current Study

The present study aimed to further understanding of factors that might counteract the occurrence of incidents of ethnic victimization. The first research question was to investigate the extent to which perceived positive contact norms in class and teachers’ reactions to ethnic victimization are linked to adolescents’ engagement in ethnicity-based negative treatments. Based on social norms theory (e.g., Perkins and Berkowitz 1986) and previous research (e.g., Schachner et al. in press; Verkuyten and Thijs 2002), it was expected that adolescents would be less likely to engage in ethnic victimization when they perceived positive contact norms in class and/or when they perceived that their teachers did not tolerate ethnicity-based victimization in class. The second research question was to examine whether the effects of perceived positive contact norms in class and teachers’ reactions to ethnic victimization on adolescents’ engagement in ethnic victimization differ across adolescents with different levels of attitudes and feelings toward immigrants. Two competing hypotheses were tested. Based on the *rich-get-richer* hypothesis (Merton 1968), it was expected that positive inter-ethnic contact norms and teachers’ clear messages regarding no tolerance of ethnic victimization would be more effective in eliminating the occurrence of ethnic victimization among adolescents who were high in positive attitudes and feelings toward immigrants. Based on the *buffering effect* hypothesis (Cohens et al. 1985), the opposite was expected. That is, social norms in class and teachers’ reactions to ethnic victimization would be more effective for adolescents who were low in positive attitudes and feelings toward immigrants.

## Methods

The sample for the current study comes from an ongoing three-year longitudinal study, the Youth and Diversity Project, which aims to examine the role of school context in the development of positive and negative interactions among adolescents of diverse backgrounds. The Youth and Diversity Project has been conducted in 16 different upper-secondary schools in four different medium-sized cities in Sweden. In each school, 7th grade students (aged around 13) were targeted. Compulsory education in Sweden starts at age 6 and continues until age 15. With some minor differences, compulsory education consists of four stages for most public-sector schools: preschool, low or primary (grades 1–3), middle or lower-secondary (grades 4–6), and high or upper-secondary (grades 7–9). Students start upper-secondary school at age 13 and graduate at age 15. The target sample consisted of 1286 adolescents (i.e., 7th grade students). Of the target sample, 17% did not participate in the study for various reasons, i.e., parents did not give consent, students themselves did not want to participate, and students were absent during data collection. A total of 1065 adolescents participated in the study; 90% of them had ethnic-victimization data. The analytical sample for the present study includes only the students with data on ethnic victimization (*N* = 963, *M*_*age*_ = 13.11, SD = 0.41; 46% girls).

A majority of the youth (72%) came from intact families and had been living with both parents (72%). More than two-thirds of the adolescents reported that their parents were working (88% of mothers, and 94% of fathers). About a quarter of them (25%) had parents who were born outside Sweden (defined as immigrant), and 13% of the youth had one parent who was born outside Sweden (defined as mixed). Among the immigrant youth, 52% were born outside Sweden (i.e., were first-generation immigrants), and only 9% reported speaking Swedish at home with their parents. About one-third of them (31%) reported speaking another language at home, and more than half (60%) reported that they sometimes spoke Swedish and sometimes another language. The immigrant adolescents’ parents had migrated to Sweden from around 60 different countries, including Iraq, Iran, Somalia, Russia, Syria, Pakistan, Turkey, Bosnia, Kosovo, Germany and Italy. Among the immigrant youth, 53% reported that they attended a native language course in school or outside school.

### Procedure

Data collection was held during two regular class hours (90 min) in the fall of 2018 across 55 classrooms in 16 different schools, and a research manager and trained research assistants supervised the data collection process. Students were informed about the goals of the study, and were assured that their participation was voluntary, and that their responses would be confidential and not be shared with anyone. Only the students whose parents did not decline their children’s participation and who themselves were willing to participate took part in the study. Each class received 500 Swedish crowns in recognition of participation, and students were provided with snacks during data collection. The Regional Research Ethics Committee in Uppsala approved the study procedures (ref. number: Dnr 2018/235).

### Measures

#### Perceived inter-ethnic contact norms and cooperation in class

The Classroom Cultural Diversity Climate scale was used to measure perceived positive inter-ethnic contact norms and cooperation in class (Schachner et al. in press). The scale includes 31 items measuring 6 different dimensions of the classroom diversity climate. In the present study, the revised version of the subscale measuring perceived inter-ethnic contact norms and cooperation in class was used. This subscale includes 5 items: “Students in my class get along well with each other even though we have different ethnic/cultural backgrounds,” “Students in my class with different ethnic backgrounds are friends with each other,” “Students in my class with different ethnic backgrounds work well together in class projects and activities,” “Students in my class are open to viewpoints different from their own,” and “Students in my class respect each other’s cultural values and customs.” Students were asked to report on how true these statements were in their classroom environment on a 5-point scale ranging from “1” (not true at all) to “5” (completely true). In the present study, Cronbach’s alpha for the five items was 0.81.

#### Teachers’ responses to ethnic victimization

A two-item measure was created as part of the project to assess adolescents’ perceptions of teachers’ responses to ethnic victimization. The items are: “Our teachers make it clear that no one can make negative comments about others because of their appearance, culture, or religion” and “Our teachers show their disapproval when they see/hear anyone making negative comments about another student because of her/his appearance, culture, or religion.” Students were asked to report on how true these statements were in their classroom environment on a 5-point scale ranging from “1” (not true at all) to “5” (completely true). These two items were positively and strongly correlated with each other (*r* = 0.52, *p* < 0.001).

#### Tolerance toward immigrants

Tolerance was conceptualized as a two-dimensional construct that includes positive attitudes and feelings toward immigrants in the present study. To measure *adolescents’ attitudes toward immigrants*, the Tolerance and Xenophobia scale was used (van Zalk et al. 2013). The scale consists of 6 items, with a sample item including: “Immigrants should have the same social rights as people born in Sweden.” Adolescents were asked to report the extent to which they agreed or disagreed with the statements on a 5-point Likert scale ranging from “1” (strongly disagree) to “5” (strongly agree). Previous research has provided evidence on the internal consistency and predictive validity of the scale (e.g., van Zalk et al. 2013). In the present study, Cronbach’s alpha was 0.84. To measure *adolescents’ feelings toward immigrants*, a revised version of the General Evaluation Scale was used (Wright et al. 1997). Adolescents were presented with the following 3 bipolar adjective pairs separated by a 7-point scale: negative–positive, hostile–friendly, and suspicious–trusting. Then, they were asked to rate the extent to which they had these feelings about immigrants without thinking of any specific person. The adolescents’ scores were averaged, with higher scores referring to greater positive feelings. In the present study, Cronbach’s alpha for the scale was 0.89.

It should be also noted that two alternative CFA models were tested and compared to investigate whether “attitudes toward immigrants” and “feelings toward immigrant” refer to empirically distinct constructs. The first model was a two-factor model where the indicators of the positive attitudes toward immigrants and positive feelings toward immigrants loaded onto their respective latent constructs. This model yielded good fit, *χ*^2^(23) = 41.27, *p* = 0.01, CFI = 1.00, RMSEA = 0.03, *p* = 0.995, 90% CI: 0.01, 0.04, SRMR = 0.018. Next, a single factor model was fitted, and this model yielded a poor fit to the data, *χ*^2^(24) = 1517.94, *p* < 0.001, CFI = 0.74, RMSEA = 0.25, *p* < 0.001, 90% CI: 0.24, 0.27, SRMR = 0.10. Comparison between the two alternative CFA models showed that the two-factor model fit the data better than the single factor model, ∆*χ*^2^(1) = 1476.67, *p* < 0.001. Overall, the results suggested that attitudes and feelings toward immigrants are interrelated, but empirically distinct constructs.

#### Engagement in ethnic victimization

A four-item scale was developed, based on a previous study (Bayram Özdemir and Stattin 2014) to measure youth’s engagement in ethnic victimization (i.e., being a perpetrator of ethnic victimization). Adolescents were asked to report on whether they had engaged in any of the behaviors at school referred to in the following questions: “Have you said nasty things to anyone about her/his ethnic origin?” “Have you excluded anyone from an activity because her/his parents came from another country?” “Have you made fun of anyone in school just because her/his parents came from another country?” “Have you avoided making friends with anyone at school because s/he or her/his parents came from another country?” Adolescents were asked to respond to each question on a 5-point scale ranging from “1” (have not done that) to “5” (several times a week). In order to test the factor structure of the ethnic victimization scale, we estimated a confirmatory factor analysis (CFA). The CFA model with 4 indicators revealed a perfect fit, *χ*^2^(2) = 3.33, *p* = 0.189, CFI = 1.00, RMSEA = 0.03, SRMR = 0.01. The standardized loadings vary between 0.68 and 0.83. The scale showed strong internal consistency (*α* = 0.88). The scores on the scale were recoded as 0 “No engagement in victimization” and 1 “Engagement in ethnic victimization at least once.” About 20% of the adolescents reported that they had engaged in ethnic victimization at least once.

#### Parental employment status

Two items were used to assess family socioeconomic status. The questions and response options were: “Does your mother work?” (0 = No, 1 = Yes); “Does your father work?” (0 = No, 1 = Yes). Using adolescents’ responses to these two questions, a parental employment variable was created with three levels: (0) neither of the parents are employed; (1) only one parent is employed; (2) both parents are employed.

#### Classroom ethnic composition

The percentage of students of Swedish background (i.e., having both parents born in Sweden) in each classroom was calculated to measure classroom ethnic composition. The proportion ranged from 0% to 88% across 55 different classrooms.

### Data Analysis

The current data included observations nested in classrooms. Thus, first, the variation in the outcome variable (i.e., ethnic victimization) across classrooms was estimated to determine whether there was a need to fit multilevel models to test the research questions (Hox 2002). A generalized linear mixed-effects model was fitted to partition the variance of ethnic victimization at individual and classroom level (Heck et al. 2010). The results showed that 6.7% of the variation in ethnic victimization was between classrooms, and the design effect (=2.11) exceeded the recommended cut off value of 2 (Muthen and Satorra 1995). Accordingly, a series of generalized linear mixed-effects models were fitted to test the research questions using Mixed Models in SPSS. Youth age, gender, immigrant background, parental employment status, and classroom ethnic composition were included in all models as control variables. Across the study variables, the proportion of missing data ranged between 0% and 6.5%, with an average of 2.2. Thus, the expectation–maximization method was employed to estimate missing data (Enders 2010).

## Results

### Descriptive Statistics and Preliminary Analysis

Means, standard deviations and correlations among the study variables are presented in Table 1. As expected, males reported engaging in ethnic victimization more than females. Immigrant adolescents (compared to Swedish adolescents) and those in classrooms with large numbers of immigrant students were more likely to engage in ethnic victimization. Adolescents with high positive attitudes and feelings toward immigrants were less likely to engage in ethnic victimization. There was also a significant negative association between adolescents’ perception of classroom social climate and their engagement in ethnic victimization, such that adolescents who reported having high positive contact norms in class were less likely to engage in ethnic victimization. Importantly, the findings also showed that when adolescents perceived that their teachers reacted to ethnic victimization, they were less likely to engage in ethnic victimization.

**Table 1:** Correlations, means, and standard deviations for the study variables

	1	2	3	4	5	6	7	8	9	10	11
1. Age	–	0.09**	0.18***	−0.06*	−0.16***	−0.07*	−0.08*	−0.05	−0.09**	−0.12***	0.15***
2. Gender^a^		–	0.03	−0.01	−0.04	−0.01	−0.10**	−0.10**	−0.04	−0.03	0.23***
3. Immigrant adolescents^b^			–	−0.22***	−0.37***	−0.38***	0.11***	0.08*	−0.09**	−0.13***	0.13***
4. Mixed adolescents^b^				–	0.03	−0.02	−0.01	0.01	0.04	0.08*	−0.01
5. Parental employment					–	0.23***	−0.03	−0.04	0.08*	0.01	−0.08*
6. Classroom ethnic composition						–	−0.14***	−0.14***	0.02	−0.01	−0.10**
7. Attitudes toward immigrants							–	0.51***	0.31***	0.17***	−0.21***
8. Feelings toward immigrants								–	0.25***	0.08*	−0.22***
9. Positive contact norms in class									–	0.37***	−0.20***
10. Teachers’ responses to ethnic victimization										–	−0.11***
11. Engagement in ethnic victimization											–
Mean	13.12	0.55	0.25	0.14	1.81	0.61	3.58	5.12	3.88	4.02	0.20
SD	0.41	0.50	0.44	0.34	0.48	0.18	0.78	1.40	0.65	0.89	0.40
Min	12	0	0	0	0	0	1	1	1	1	0
Max	15	1	1	1	2	0.88	5	7	5	5	1

^a^Gender was coded as: “0” girls and “1” boys

^b^“Swedish adolescents” was defined as reference category

*p < 0.05; **p < 0.01; ***p < 0.001

### Perceived Positive Contact Norms and Cooperation in Class

Two generalized linear mixed-effects models were fitted to examine: (1) whether there was a unique association between adolescents’ perception of positive contact norms in class and their engagement in ethnic victimization, and (2) whether any such association was moderated by adolescents’ attitudes and feelings toward immigrants. Adolescents’ age, gender and immigrant status, parental employment, and classroom ethnic composition were all controlled for (see Tables 2 and 3). First, a two-level model, where classroom ethnic composition and positive contact norms in class were entered as level-2 predictors was fitted. The Hessian matrix was non-positive definite due to low variance at the second level. Thus, level-2 variations were fixed at zero (Heck et al. 2010). The results of generalized linear mixed-effects models showed that older adolescents, boys and adolescents of migrant background had a greater likelihood of engaging in ethnic victimization than girls and native Swedish adolescents, respectively. Positive attitudes and feelings toward immigrants were likely to lower the likelihood of engagement in ethnic victimization. In addition, students who reported more positive contact norms in class were less likely to ethnically victimize their peers (*b* = –0.53, SE = 0.16, *t* = −3.50, *p* < 0.001, OR = 0.60). Neither adolescents’ attitudes toward immigrants (*b* = −0.17, SE = 0.15, *t* = −1.22, *p* = 0.225, OR = 0.85) nor their feelings toward immigrants (*b* = −0.16, SE = 0.10, *t* = −1.70, *p* = 0.091, OR = 0.86) significantly moderated the association between positive contact norms in class and engagement in ethnic victimization. Together, these findings suggest that being in an inclusive and socially cohesive classroom environment may have the potential to reduce engagement in ethnic victimization similarly across youth at different levels with regard to their attitudes and feelings toward immigrants.

**Table 2:** Association between positive contact norms in class and adolescents’ engagement in ethnic victimization: do adolescents’ attitudes matter?

						95% CI OR	
	*b*	SE	*t*	*p*	OR	LL	UP
Intercept	−2.64	0.21	−12.63	<0.001	0.08	0.05	0.11
Age	0.55	0.22	2.49	0.013	1.72	1.13	2.64
Gender^a^	1.30	0.21	6.33	<0.001	3.66	2.45	5.47
Immigrant adolescents^b^	0.69	0.24	2.88	0.004	2.00	1.25	3.20
Mixed adolescents^b^	0.32	0.28	1.14	0.257	1.38	0.80	2.38
Parental employment	0.05	0.19	0.22	0.828	1.05	0.72	1.52
Classroom ethnic composition	−1.09	0.59	−1.86	0.064	0.34	0.11	1.07
Attitudes toward immigrants (AI)	−0.37	0.14	−2.78	0.006	0.70	0.54	0.90
Feelings toward immigrants (FI)	−0.24	0.08	−3.29	<0.001	0.79	0.69	0.91
Positive contact norms in class (PCN)	−0.51	0.16	−3.33	0.001	0.61	0.46	0.82
AI × PCN	−0.17	0.15	−1.22	0.225	0.85	0.65	1.12

The level-2 variance component in the model was estimated initially; however, the Hessian matrix was not positive definite due to lack of variation between classrooms. Therefore, the level-2 variance component was set at zero and the model re-estimated

^a^Gender was coded as: “0” girls and “1” boys

^b^“Swedish adolescents” was defined as reference category

**Table 3:** Association between positive contact norms in class and adolescents’ engagement in ethnic victimization: do adolescents’ feelings toward immigrants matter?

						95% CI OR	
	*b*	SE	*t*	*p*	OR	LL	UP
Intercept	−2.66	0.21	−12.66	<0.001	0.08	0.05	0.11
Age	0.58	0.22	2.65	0.008	1.78	1.16	2.73
Gender^a^	1.32	0.21	6.40	<0.001	3.73	2.49	5.59
Immigrant adolescents^b^	0.69	0.25	2.87	0.004	2.00	1.25	3.20
Mixed adolescents^b^	0.29	0.28	1.03	0.303	1.34	0.78	2.31
Parental employment	0.06	0.19	0.32	0.751	1.07	0.74	1.54
Classroom ethnic composition	−1.16	0.59	−1.98	0.049	0.32	0.10	1.00
Attitudes toward immigrants (AI)	−0.37	0.14	−2.74	0.006	0.70	0.54	0.91
Feelings toward immigrants (FI)	−0.26	0.08	−3.52	<0.001	0.78	0.67	0.90
Positive contact norms in class (PCN)	−0.53	0.16	−3.50	<0.001	0.60	0.45	0.80
FI × PCN	−0.16	0.10	−1.70	0.091	0.86	0.72	1.03

The level-2 variance component in the model was estimated initially; however, the Hessian matrix was not positive definite due to lack of variation between classrooms. Therefore, the level-2 variance component was set at zero and the model re-estimated

^a^Gender was coded as: “0” girls and “1” boys

^b^“Swedish adolescents” was defined as reference category

### Perceived Teachers’ Responses to Ethnic Victimization

Two generalized linear mixed-effects models were estimated to examine: (1) whether there was a unique association between adolescents’ perceptions of teachers’ reactions and their engagement in ethnic victimization, and (2) whether the association was moderated by adolescents’ attitudes and feelings toward immigrants. Adolescents’ age, gender, and immigrant status, parental employment, and classroom ethnic composition were all controlled for in these regression models. Similar to the previous generalized linear mixed-effects models, the Hessian matrix was non-positive definite due to low variance at the second level when classroom level variables were modeled as level-2 covariates. Thus, level-2 variations were fixed at zero (Heck et al. 2010) (see Tables 4 and 5).

**Table 4:** Association between teachers’ responses to ethnic victimization and adolescents’ engagement in ethnic victimization: do adolescents’ attitudes matter?

						95% CI OR	
	*b*	SE	*t*	*p*	OR	LL	UP
Intercept	−2.65	0.22	−12.58	<0.001	0.08	0.05	0.11
Age	0.55	0.22	2.52	0.012	1.72	1.13	2.63
Gender^a^	1.33	0.21	6.43	<0.001	3.76	2.51	5.63
Immigrant adolescents^b^	0.68	0.25	2.82	0.005	1.97	1.23	3.17
Mixed adolescents^b^	0.33	0.29	1.16	0.248	1.39	0.80	2.41
Parental employment	0.04	0.20	0.22	0.833	1.05	0.72	1.52
Classroom ethnic composition	−1.19	0.60	−2.00	0.046	0.31	0.10	0.98
Attitudes toward immigrants (AI)	−0.46	0.14	−3.40	0.001	0.64	0.49	0.83
Feelings toward immigrants (FI)	−0.26	0.08	−3.59	<0.001	0.78	0.67	0.89
Teachers’ responses to ethnic victimization (TREV)	−0.19	0.11	−1.81	0.071	0.83	0.68	1.02
AI × TREV	−0.29	0.11	−2.78	0.006	0.75	0.62	0.92

The level-2 variance component in the model was estimated initially; however, the Hessian matrix was not positive definite due to lack of variation between classrooms. Therefore, the level-2 variance component was set at zero and the model re-estimated

^a^Gender was coded as: “0” girls and “1” boys

^b^“Swedish adolescents” was defined as reference category

**Table 5:** Association between teachers’ responses to ethnic victimization and adolescents’ engagement in ethnic victimization: do adolescents’ feelings toward immigrants matter?

						95% CI OR	
	*b*	SE	*t*	*p*	OR	LL	UP
Intercept	−2.65	0.21	−12.65	<0.001	0.08	0.05	0.11
Age	0.59	0.22	2.73	0.006	1.8	1.18	2.75
Gender^a^	1.30	0.21	6.34	<0.001	3.66	2.45	5.47
Immigrant adolescents^b^	0.68	0.25	2.81	0.005	1.97	1.23	3.16
Mixed adolescents^b^	0.33	0.29	1.17	0.244	1.39	0.80	2.41
Parental employment	0.02	0.20	0.11	0.915	1.03	0.71	1.49
Classroom ethnic composition	−1.23	0.60	−2.06	0.040	0.30	0.10	0.95
Attitudes toward immigrants (AI)	−0.39	0.14	−2.94	0.003	0.69	0.53	0.89
Feelings toward immigrants (FI)	−0.32	0.08	−4.17	<0.001	0.74	0.64	0.85
Teachers’ responses to ethnic victimization (TREV)	−0.20	0.11	−1.90	0.059	0.82	0.67	1.01
FI × TREV	−0.20	0.07	−2.93	0.004	0.83	0.73	0.94

The level-2 variance component in the model was estimated initially; however, the Hessian matrix was not positive definite due to lack of variation between classrooms. Therefore, the level-2 variance component was set at zero and the model re-estimated

^a^Gender was coded as: “0” girls and “1” boys

^b^“Swedish adolescents” was defined as reference category

**Fig. 1:**
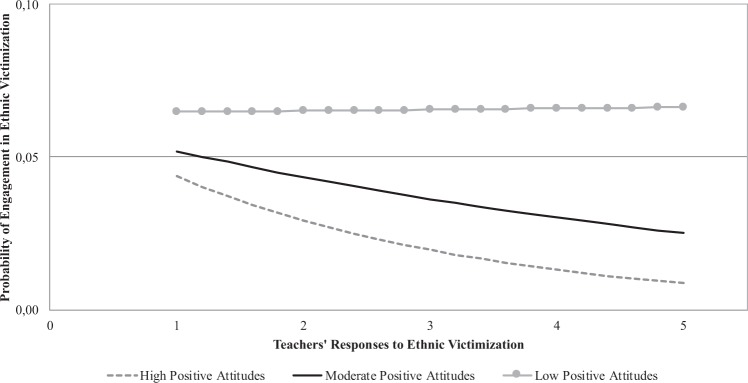
Moderating role of attitudes in the association between teachers’ responses to ethnic victimization and engagement in ethnic victimization

The results showed that teachers’ reactions to ethnic victimization were linked to a reduced likelihood of engaging in ethnic victimization, but this effect did not reach significance. On the other hand, adolescents’ attitudes toward immigrants (*b* = −0.29, SE = 0.11, *t* = −2.78, *p* = 0.006, OR = 0.75) significantly moderated the association between perceived teachers’ responses and adolescents’ engagement in the ethnic victimization of their peers. The results of simple slope testing showed that, at high levels of positive attitudes toward immigrants, perceived teachers’ reactions significantly and negatively predicted the likelihood of engagement in ethnic victimization (*b* = −0.41, SE = 0.14, *t* = −2.91, *p* = 0.004, OR = 0.66). By contrast, at moderate and low levels of positive attitudes toward immigrants, the association between teachers’ reactions and engagement in ethnic victimization was not statistically significant (*b* = −0.19, SE = 0.10, *t* = −1.81, *p* = 0.071, OR = 0.82 and *b* = 0.04, SE = 0.12, *t* = 0.31, *p* = 0.760, OR = 1.04, respectively) (see Fig. 1).

**Fig. 2:**
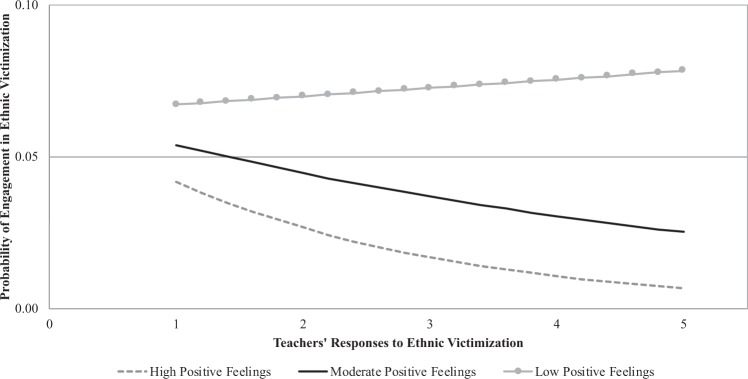
Moderating role of feelings toward immigrants in the association between teachers’ responses to ethnic victimization and engagement in ethnic victimization

Similar findings were also observed for the moderating effect of feelings toward immigrants (*b* = −0.20, SE = 0.07, *t* = −2.93, *p* = 0.004, OR = 0.83). Specifically, the results of simple slope tests showed that, at high levels of positive feelings toward immigrants, teachers’ reactions significantly and negatively predicted the likelihood of engagement in ethnic victimization (*b* = −0.47, SE = 0.15, *t* = −3.09, *p* = 0.002, OR = 0.63). However, such an association was not observed at moderate and low levels of positive feelings toward immigrants (*b* = −0.20, SE = 0.11, *t* = −1.90, *p* = 0.059, OR = 0.82, and *b* = 0.07, SE = 0.13, *t* = 0.56, *p* = 0.575, OR = 1.07, respectively) (see Fig. 2). Together, supporting the premises of the *rich-get-richer* hypothesis, these findings suggest that when teachers make it clear to students that no-one can make negative comments about others because of their background, students with positive attitudes and feelings toward immigrants are less likely to engage in ethnic victimization. However, teachers’ reactions do not have an impact on youth’s engagement in ethnic victimization at moderate and low levels of positive attitudes and feelings toward immigrants.

## Discussion

Today’s youth are growing up in ethnically diverse settings. Some youth appreciate this diversity and take the opportunity to engage with diverse perspectives, whereas others are more hesitant in interacting with others who are different from themselves, or may even engage in hostile behaviors toward them (e.g., ethnic victimization). Being exposed to ethnic victimization has detrimental consequences for young people’s psychosocial functioning and behavioral adjustment (e.g., Bayram Özdemir and Stattin 2014; Bayram Özdemir et al. 2019), and disrupts their integration into the host society (e.g., Marks et al. 2015). Despite an increasing number of studies on ethnic victimization, the bulk of existing research has adopted a victim perspective that aims at understanding the consequences of being the target of negative treatment. Relatively little attention has been paid to understanding the problem from the perpetrator’s perspective (Bayram Özdemir et al. 2019; Caravita et al. 2019; Larochette et al. 2010), or to the factors that might counteract the occurrence of ethnic victimization. The present study aimed to address these gaps in knowledge by examining the extent to which perceived positive contact norms in classrooms and teachers’ reactions to ethnic victimization play a role in adolescents’ engagement in ethnicity-based negative treatments. The present study also examined for whom perceived positive contact norms in classroom and teachers’ reactions to ethnic victimization matter most by focusing on adolescents’ attitudes and feelings toward immigrants.

One of the important contributions of the present study is that it draws attention to the importance of classroom context in understanding the factors that may contribute to adolescents’ engagement in ethnic victimization. Supporting the premises of social norms theories (Perkins and Berkowitz 1986) and previous research (e.g., Schachner et al. in press; Schwarzenthal et al. 2019), the current findings suggest that, when adolescents perceive that students in their classroom are open to diverse views, respect each other’s cultural values, and cooperate with each other on different class activities, they are less likely to engage in ethnic victimization. Importantly and interestingly, the effect of perceived positive contact norms on engagement in ethnic victimization is the same among youth with different attitudes and feelings toward immigrants, even after controlling for certain demographic factors, such as gender, immigrant status, and classroom ethnic composition. That is, the likelihood of engagement in ethnic victimization becomes lower for both adolescents who are at low risk (i.e., those high in positive attitudes and feelings) and those who are at high risk (i.e., those low in positive attitudes and feelings). This finding indicates that an optimal social context (i.e., perceived positive social contact norms in class, in this case) might be beneficial for adolescents with high social capital as well as for adolescents who are at risk.

Two alternative explanations can be proposed for this finding. As previously mentioned, being in an inclusive and socially cohesive classroom may contribute to the development of intercultural competence and awareness among adolescents (Schwarzenthal et al. 2019) and foster cross-ethnic friendships (Schachner et al. 2015). Such competence and inter-personal relationships may help adolescents embrace differences rather than perceive them as a threat, and, in turn, may prevent the occurrence of incidents of ethnic victimization. Alternatively, when adolescents perceive that inclusion and social cohesion are the norm in their classroom, they may be hesitant to victimize their peers due to their background because they fear negative reactions from the rest of their class. That is, in order to avoid social sanctions, they may act in line with prevailing classroom norms.

Another important contribution of the present study lies in its examination of whether teachers’ reactions to ethnic victimization have an impact on youth’s behaviors, and for whom such reactions are most effective. In line with previous research (Verkuyten and Thijs 2002), the current findings show that when teachers make it clear to students that no-one can make negative comments about others because of their background, adolescents (on average) are less likely to engage in ethnic victimization. Importantly, the findings also reveal that not all adolescents are affected similarly by teachers’ reactions; that is, there is clear heterogeneity. Specifically, it was found that when adolescents hold high levels of tolerance toward immigrants, teachers’ clear messages of no tolerance of victimization are negatively associated with engagement in ethnic victimization. However, such teacher reactions do not affect the behaviors of adolescents of low and moderate tolerance toward immigrants. This finding supports the *rich-get richer* hypothesis (Merton 1968), and indicates that teachers’ messages of no-tolerance of ethnic victimization are effective for young people who are already at low risk of engagement in ethnic victimization but not for those who are at high risk. As discussed previously, teachers’ no-tolerance strategies are in line with the views and feelings of tolerant adolescents, and may validate these adolescents’ views on diversity. Thus, it might be a lot easier for them to internalize the rules that teachers endorse.

Then, the question arises why teachers’ messages of no tolerance of ethnic victimization do not have an impact on youth with low positive attitudes and feelings toward immigrants. One possible explanation is that such a teachers’ approach might not be enough to address the roots of victimization and to provide youth at risk with the skills and resources that would help them develop healthy social interactions. Rather, the approach might lead intolerant adolescents to experience a clash between their personal views on immigrants and the rules endorsed in school. This perception of a clash might prevent these youth from internalizing teachers’ messages. Thus, in addition to adopting a no-tolerance approach, teachers might need to implement strategies to help intolerant youth *in*validate their prejudiced views and negative feelings about immigrants, and help them develop the empathy and perspective-taking skills that, for instance, have been shown to prevent the occurrence of bullying (see van Noorden et al. 2015 for a meta-analytical review) and discrimination (Abbott and Cameron 2014). Such a broad approach may have the potential to foster healthy social interactions among adolescents of diverse backgrounds.

The results also reveal that adolescents of immigrant background are more likely to engage in ethnic victimization than their native counterparts. This finding is consistent with previous research findings by Larochette et al. 2010, which have shown that minority students (in particular African-Canadians) engage in more racial bullying and victimization than their European-Canadian counterparts. One possible explanation for this finding may be related to the extent to which immigrant and Swedish youth are differentially exposed to peers of foreign background. As in other European countries, immigrants in Sweden tend to live in segregated neighborhoods, which are populated by people who have come from other countries. Relatedly, immigrant adolescents have a higher likelihood of being exposed to peers of diverse backgrounds in school than their Swedish counterparts. The greater mutual physical proximity of immigrant youth may be one of the underlying reasons why immigrant youth are more at risk of engagement in ethnic victimization than their Swedish counterparts (Blau 1977). More generally, this finding also highlights an inherent limitation of the tendency to focus on native youth in studies that aim to understand the prevalence and predictors of ethnic/racial bullying and victimization (e.g., Bayram Özdemir et al. 2019; Bayram Özdemir et al. 2016). Understanding precursors of ethnic victimization of majority youth toward minority and immigrants may guide practices and programs that aim to prevent negative encounters among diverse youth. But there is also a need to develop a comprehensive understanding of why immigrant youth are more likely to engage in ethnic victimization, and of the underlying aspects of ethnic victimization that are both common to and distinct between immigrant and native adolescents.

The findings also show that boys have a greater likelihood of engaging in ethnic victimization, which is consistent with previous research (Bayram Özdemir et al. 2018; Larochette et al. 2010). One explanation for the gender difference may be related to the differences in emotional and cognitive skills between males and females. Specifically, the literature consistently shows that females are better at perspective-taking (e.g., Tucker Smith et al. 2016) and have greater empathic concerns (e.g., Butrus and Witenberg 2013). These advanced emotional and cognitive skills may help females become better aware of the possible consequences of victimization for its targets than males, and, in turn, dissuade them from engaging in ethnic victimization (Topcu and Erdur-Baker 2012). In sum, males’ tendency to engage in ethnic victimization more than females may be due to differences in perspective-taking skills or empathic concerns rather than gender per se. This conceptual explanation requires further examination in future research.

Despite its important contributions to the literature, several limitations of the present research need to be acknowledged. First, the study presented here was correlational by nature, and the data captured only one time-point. The inherent limitations of cross-sectional data limit the possibility of examining whether and for whom perceived contact norms in class and teachers’ reactions have an impact on changes in adolescents’ engagement in ethnic victimization over time. The correlational nature of the data also limits the capacity to examine the directionality of the effects. In the current study, ethnic victimization was conceptualized as an outcome and perceived contact norms in class and teachers’ reactions as predictors. Adolescents who engage in ethnic victimization might alter their perception of contact norms in class, and also teachers’ reactions, to alleviate potential stress due to cognitive dissonance. Alternative models could be tested using experiential sampling methods that allow researchers to examine day-to-day changes in targeted outcomes and potential explanations for these changes. Second, the current study relied on youth’s self-reports in the measurement of the study’s constructs. Such reliance raises two concerns. First, it is not possible to determine whether adolescents’ perceptions of teachers’ behaviors accurately reflect the actual behaviors of teachers. Second, relying heavily on self-report measures may have inflated the associations between variables in the models due to common method variance. Thus, future studies using multiple informants may advance the literature on ethnic victimization. Third, in the present study, only one aspect of teachers’ reactions to ethnic victimization, so-called no-tolerance, was in focus. However, a growing body of literature has shown than teachers may use multiple strategies to handle problematic behaviors (Bauman et al. 2008), such as enlisting the parents of victims and perpetrators, discussing cooperative actions with other teachers, empowering, comforting and supporting the victims individually, and building empathy and perspective-taking skills among bullies. A comprehensive understanding of how these different strategies have an impact on the perpetrators of ethnic victimization is needed.

## Conclusion

The present study sheds light on the factors that may deter youth from victimizing their peers due to their ethnic, cultural, or religious backgrounds. The findings suggest that, when adolescents perceive that students in their classroom are open to diverse views, respect each other’s cultural values, and cooperate with each other on different class activities, they are less likely to engage in ethnic victimization, regardless of their views on and feelings about immigrants. This finding highlights the importance of establishing a classroom context where diverse views and values are appreciated and respected, and where adolescents cooperate on day-to-day activities in order to prevent incidents of ethnic victimization. The findings also suggest that teachers’ clear messages of no tolerance of ethnic victimization have the potential to deter youth from victimizing their peers, but only those youth who already have positive attitudes and feelings toward immigrants. Unfortunately, youth who are intolerant of and feel negatively about their immigrant peers are still at risk of engaging in ethnic victimization even when they are aware of their teachers’ no-tolerance attitude. Thus, in addition to adopting a no-tolerance approach, other strategies in schools to target at-risk adolescents (i.e., prejudiced youth) more effectively are warranted. To conclude, schools and teachers may have the possibility to counteract negative interactions among students of diverse backgrounds through how they “*Walk the Talk*”.
